# False Recognition of Emotionally Categorized Pictures in Young and Older Adults

**DOI:** 10.3389/fpsyg.2019.01477

**Published:** 2019-06-27

**Authors:** Zhiwei Zheng, Minjia Lang, Wei Wang, Fengqiu Xiao, Shuhan Guo, Juan Li

**Affiliations:** ^1^Center on Aging Psychology, CAS Key Laboratory of Mental Health, Institute of Psychology, Chinese Academy of Sciences, Beijing, China; ^2^Department of Psychology, University of Chinese Academy of Sciences, Beijing, China; ^3^China National Children’s Center, Beijing, China; ^4^Magnetic Resonance Imaging Research Center, Institute of Psychology, Chinese Academy of Sciences, Beijing, China; ^5^State Key Laboratory of Brain and Cognitive Science, Institute of Biophysics, Chinese Academy of Sciences, Beijing, China

**Keywords:** positivity effect, false memory, emotion regulation, executive functioning, aging

## Abstract

Normal aging is associated with the shift in motivational priorities from knowledge acquisition to emotion regulation. Current evidence indicates an age-related increase in preferences for positive over negative stimuli in true memory. In the present study, using the categorized pictures paradigm, we investigated whether older adults would exhibit a greater increase in false memory for positive versus negative lures, compared with young adults. We also examined the association of executive functioning with the preferences for positive over negative pictures in false recognition memory. A total of 27 young and 26 older adults studied emotional pictures from various categories during encoding and subsequently completed an old/new recognition test. In addition, all participants completed the executive functioning tests. The results revealed that both older and young adults showed higher rates of false recognition for positive pictures compared with negative pictures; no significant group by valence interaction was observed. Trail making scores were negatively correlated with positive processing preferences in false recognition rates in older but not young adults. These findings suggest that false recognition memory exhibits preferences toward positively valenced stimuli in both young and older adults. Cognitive control processes are necessary for older adults to distort memory in emotionally gratifying ways.

## Introduction

It is well established that emotionally arousing events are generally remembered better than neutral ones (Hamann, 2001; Talmi, 2013). In laboratory studies, the beneficial effects of emotion on episodic memory have been confirmed with a variety of stimuli, including pictures (e.g., Hamann et al., 1999), words (e.g., Phelps et al., 1997), and stories (e.g., Cahill and McGaugh, 1995). This phenomenon of enhanced memory for emotional events is well consistent with the evolutionary perspective, which suggests that emotional stimuli may contain more survival-relevant information and is important for individuals to facilitate avoidance and reproductive success when they re-encounter similar events in the future (Ochsner, 2000; Dolan, 2002). Cognitive neuroscience studies demonstrate that interactions between the amygdala and other brain regions such as the hippocampus and prefrontal cortex (PFC) are critical for the storage and retrieval of emotional memory (McGaugh, 2004; Buchanan, 2007).

As people age, they generally exhibit a profound decline in episodic memory (Shing et al., 2010). Nevertheless, the memory benefits for emotional materials are preserved across the adult lifespan. Like young adults, older adults are more likely to remember emotional events than neutral ones (Kensinger et al., 2014). However, there may be age differences in episodic memory for emotionally valenced stimuli. For example, accumulating evidence demonstrates an age-related positivity effect^1^ in episodic memory (Carstensen and Mikels, 2005; Mather and Carstensen, 2005; Reed and Carstensen, 2012; Reed et al., 2014). Specifically, compared to young adults, older adults remember more positive versus negative information for autobiographical events (Kennedy et al., 2004), emotional pictures (Charles et al., 2003), faces (Mather and Carstensen, 2003), and words (Kensinger, 2008). These findings can be interpreted according to socioemotional selectivity theory (Carstensen et al., 1999; Carstensen, 2006; Reed and Carstensen, 2012), which proposes that human motivational priorities shift as people age from acquiring knowledge to regulating emotion to attain better emotional well-being. Thus, the positivity effect in older adults may be attributed to their prioritization of emotional regulation goals.

In addition to investigating emotional memory for studied events, a few studies have attempted to provide evidence for age differences in emotional memory by examining false memory. For example, Fernandes et al. (2008) offered support for the age-related positivity effect in a free-recall test. Specifically, they reported that older but not young participants consistently falsely recalled more positive versus negative information across three types of material (i.e., autobiographical events, pictures, and words). Interestingly, in another study using a cued-recall task during which participants were asked to indicate whether the test labels were presented together with a picture during the study phase (Gallo et al., 2009), both young and older adults showed similar proportions of false recollection judgments between positive and negative conditions. Finally, using the Deese-Roediger-McDermott (DRM) paradigm (Deese, 1959; Roediger and McDermott, 1995), Piguet et al. (2008) reported that older relative to young adults showed more false recognition rates to positive than negative lures that were orthographically related to the neutral words presented during encoding. Taken together, the previous studies have provided mixed findings on the age-related positivity effect in false memory.

One plausible explanation for these inconsistent findings is that different cognitive processes are involved in the false memory tasks utilized in previous studies. The free- and cued-recall tasks are primarily designed to examine the effects of recollection process on the production of false memory (Fernandes et al., 2008; Gallo et al., 2009). However, older adults may sometimes fail to implement their emotional regulation goals on recollection process (e.g., Gallo et al., 2009), since it is widely believed that normal aging is associated with impairments in recollection (Koen and Yonelinas, 2014). As a result, the inconsistent observations on the age-related positivity effect in false recall may be due to their impaired recollection process in older adults. It may be not feasible to use recall paradigm to assess age-related positivity effect in false memory.

The DRM task is one of the most widely used paradigms to examine false recognition memory. With regard to cognitive processes engaged in false recognition, the activation-monitoring theory suggests that the representations of nonpresented lures semantically related to study items are strongly activated, and can be bound with contextual details during the study phase (Roediger et al., 2001). Thus, recollection process can greatly support false recognition because contextual details are incorrectly retrieved during test (see Arndt, 2012 for a review). On the contrary, the fuzzy-trace theory mainly emphasizes the contribution of familiarity to false recognition memory. Specifically, this perspective suggests that gist memory traces of study items created during encoding can support the production of false memory based on familiarity process during test (Brainerd and Reyna, 2002, 2005; Bookbinder and Brainerd, 2016). It is well established that familiarity is relatively preserved in older adults (Koen and Yonelinas, 2014). Following this logic, it is reasonable to speculate that false recognition tasks may be better to uncover the age-related positivity effect, because older adults may be more likely to implement their emotional regulation goals on familiarity process. To date, only Piguet et al. (2008) examined the age differences in emotional false recognition memory in a DRM task. They did reveal that older adults showed higher false recognition rates to positive than negative lures. However, it should be noted that all the study items were neutral (e.g., hear, near, clear, gear), and emotional lures (e.g., cheer) were orthographically related to the study items in their false memory task. As such, the false recognition performance of emotionally valenced lures may be confounded by the conceptual incongruence of emotional lures with the study items, rather than reflecting the emotional effects of these lures.

In the present study, we aimed to further examine whether older adults would exhibit a positivity effect in false recognition memory. Specifically, in a categorized pictures paradigm, young and older participants encoded emotional and neutral pictures from various categories and then performed an old/new recognition memory task that included old pictures, lure pictures categorically related to the old pictures, and new pictures. The categorized pictures task was used based on two considerations. On the one hand, as a modified version of the DRM task, it is also a widely used paradigm to examine false recognition memory (e.g., Budson et al., 2006; Gutchess and Schacter, 2012). On the other hand, in this task, participants were presented with the same number of emotional and neutral stimuli during encoding, thus ruling out the possibility of potential conceptual incongruence between the emotional lures and the study items contributing to the increased false recognition rates of positive lures.

Normal aging is associated with increased susceptibility to memory distortions (e.g., Koutstaal and Schacter, 1997; Tun et al., 1998; Balota et al., 1999). The findings of age-related increases in false memory have been interpreted as reflecting older adults’ overreliance on gist processing during encoding and familiarity process during retrieval (Devitt and Schacter, 2016). If emotion regulation goals are prioritized for older adults, they may be more likely to create strong gist memory traces for positive items and subsequently falsely endorse the positive lures as old items based on enhanced familiarity-based retrieval in a goal-consistent manner. This speculation is consistent with the finding that older adults experience a greater feeling of familiarity for positive stimuli compared with younger adults in recognition memory (Spaniol et al., 2008). As a result, in the present study, we expected that older participants would exhibit a preference for positive over negative lure pictures in their false recognition memory. Accordingly, they would show higher false recognition rates for emotionally positive lures relative to negative ones.

In addition, we examined individual differences in older adults’ positive processing preferences in false recognition memory. It has been suggested that executive functioning is necessary for the emergence of age-related positivity effect (Mather and Carstensen, 2005; Reed and Carstensen, 2012). According to socioemotional selectivity theory, older adults with higher executive functioning are more likely to exhibit the positivity effect, as they have more cognitive control resources to implement their emotional regulation goals by strengthening positive and diminishing negative stimuli in memory (Mather, 2006). Consistently, Mather and Knight (2005) reported that older adults with high scores on executive functioning tests remembered high proportions of positive than negative pictures, whereas the recall proportions of negative pictures were higher than positive pictures in older adults with low executive functioning scores. In addition, older adults’ scores on an executive attention task could predict their subjective experiences associated with the retrieved memories for positive rather than negative life events (Petrican et al., 2008). That is, older adults with higher executive functioning have richer subjective recollective experiences in their memories for positive public events. These findings suggest that older adults’ executive functioning levels may modulate their preferences for positive over negative information in memory. In the present study, we analyzed the correlations between the scores on the neuropsychological tests of executive functioning and positive processing preferences in false recognition rates. We expected that older adults with higher executive functioning would be more likely to show greater preferences for positive over negative lure pictures in false recognition memory.

## Materials and Methods

### Participants

A total of 27 right-handed young adults (age range: 19–25 years) and 26 older adults (age range: 61–79 years) participated voluntarily in this study and received monetary compensation for their participation. G*Power (v. 3.1; Faul et al., 2007) was used to determine sample size. *A priori* power analysis showed that 17 participants per group would yield 80% power to detect a Group × Valence interaction in false memory, given a medium effect size of *f* = 0.229 (Fernandes et al., 2008), and a significance level of 5%. However, for counterbalancing purposes, a larger sample size was used. With our sample of 27 young and 26 older adults, the statistical power of achieving an effect of *f* = 0.229 is higher than 95% for detecting the Group × Valence interaction in false memory.

The demographic characteristics of the participants are presented in Table 1. Young and older adults were equated on gender distribution and education levels. Young adults were college students recruited online from universities in Beijing. Older adults were community dwellers recruited from the research participant pool at the Institute of Psychology, Chinese Academy of Sciences. All participants reported that they were healthy, native Chinese speakers, free from psychiatric or neurological disorders, and had normal or corrected-to-normal vision. The experimental procedures were approved by the Ethics Committee of the Institute of Psychology, Chinese Academy of Sciences, and all participants provided informed consent prior to participation.

**Table 1:** Participant characteristics and neuropsychological test performance for each age group (mean and standard deviations).

	Young (*n* = 27)	Older (*n* = 26)	*p*
Age (years)	22.481 (1.626)	69.462 (5.014)	–
Education (years)	14.815 (1.178)	14.538 (2.533)	0.616^a^
Gender (M/F)	14/13	15/11	0.669^b^
MoCA^c^	–	27.615 (1.878)	–
Trail Making A (s)	24.843 (6.880)	36.204 (11.278)	<0.001^a^
Trail Making B (s)	32.701 (7.807)	72.538 (38.124)	<0.001^a^
Digit Span Forward	9.407 (2.390)	7.385 (1.651)	0.001^a^
Digit Span Backward	7.037 (1.652)	5.346 (1.263)	<0.001^a^
Block design test	41.852 (5.433)	30.192 (7.294)	<0.001^a^
Logic memory-immediate	11.407 (1.721)	8.808 (2.164)	<0.001^a^
Logic memory-delayed	10.241 (1.547)	7.288 (2.201)	<0.001^a^
Paired-association learning	5.167 (1.906)	2.423 (1.369)	<0.001^a^
Vocabulary test	63.889 (5.632)	63.769 (7.659)	0.948^a^
Category fluency test	24.852 (5.318)	24.192 (7.381)	0.710^a^

a An independent samples two-tailed *t*-test.

b A two-tailed Pearson chi-square test.

c Montreal cognitive assessment.

### Neuropsychological Assessment

All participants completed a battery of neuropsychological tests at the end of the experimental session. The older participants were first given the Montreal Cognitive Assessment-Beijing Version as a preliminary screening measure for mild cognitive impairment with a cutoff score of 21/30 (Yu et al., 2012). The battery was composed of the following: Trail Making Tests A and B, assessing executive functions (Reitan, 1992); Digit Span Forward and Digit Span Backward, indexing working memory (Wechsler, 1981); Block Design Test, indexing visuospatial ability (Wechsler, 1981); Paired-Association Learning Test from the Clinical Memory Scale (Xu and Wu, 1986) and Logic Memory Immediate and Delayed Recall (Wechsler, 1987), assessing long-term memory; and Vocabulary Test (Wechsler, 1981) and Category Fluency Test (Spreen and Strauss, 1998), indexing semantic memory. These screening tests ensured that older participants’ fluid intelligence and crystallized intelligence were in the normal range.

### Materials

**Figure 1:**
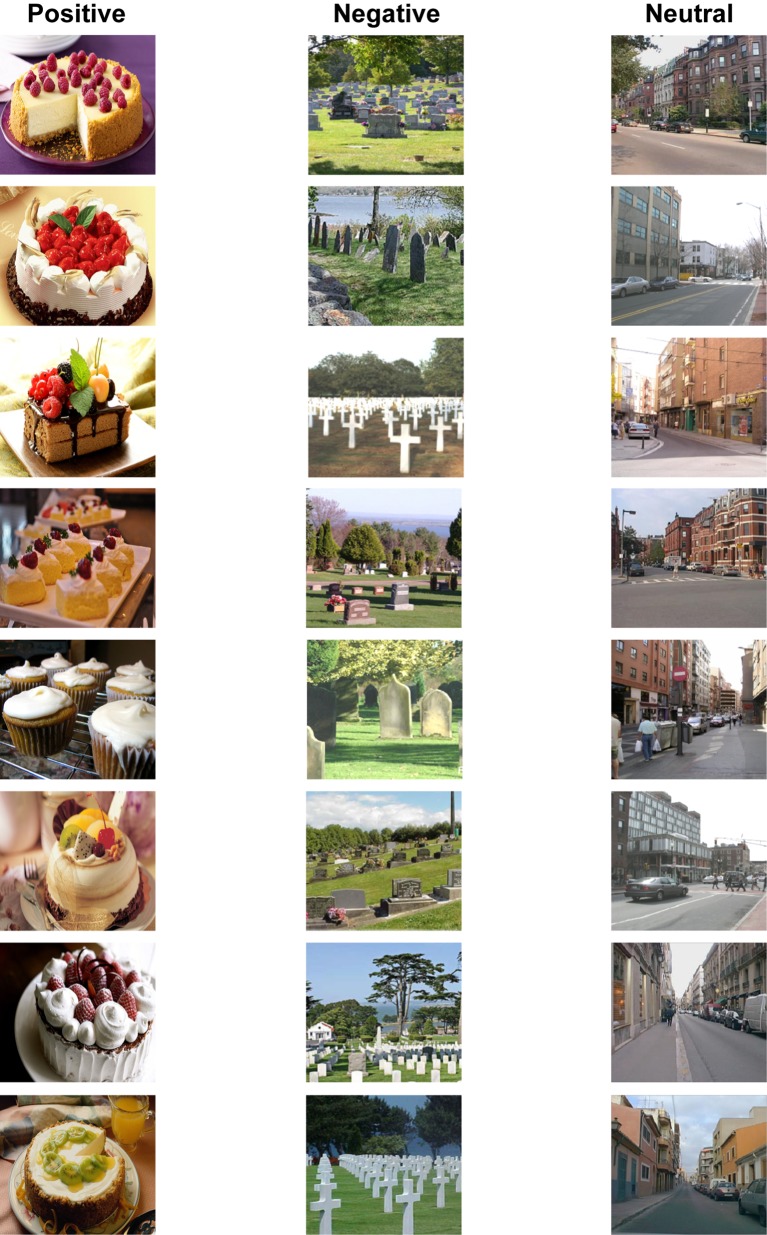
Examples of experimental stimuli. There were 27 positive, 27 neutral, and 27 negative categories with eight typical exemplars per category. For each studied category, four exemplars were randomly presented at study and then served as old items at test; the remaining four exemplars were presented as lures at test. For each unstudied category, all exemplars were presented as new items at test.

A total of 648 realistic pictures consisting of 81 categories were obtained from the International Affective Picture System (Lang et al., 2008) and Internet searches^2^. There were 27 positive (e.g., cakes), 27 neutral (e.g., street scenes), and 27 negative (e.g., graves) categories, with each category consisting of eight typical exemplars. All pictures were resized to a size of 300 × 270 pixels (see Figure 1) and digitally matched for brightness and contrast.

The valence (negative/positive) and arousal (calm/aroused) of the pictures were assessed based on the Self-Assessment Manikin Scale (Bradley and Lang, 1994). Ten young adults (6 males, *M_age_* = 22.2 years, *M*_education_ = 14.5 years) and 10 older adults (5 males, *M_age_* = 68.8 years, *M*_education_ = 15.3 years), none of whom subsequently participated in the formal memory experiment, were recruited for this rating using a 9-point scale ranging from 1 = extremely unpleasant to 9 = extremely pleasant for valence and from 1 = extremely calm to 9 = extremely aroused for arousal. The 3 (valence: positive vs. neutral vs. negative) × 2 (group: young vs. older) repeated measures analysis of variance (ANOVA) was separately conducted on rating results of valence and arousal. The analysis on rating results of valence only revealed a significant main effect of valence, *F*(2, 36) = 133.56, *p* < 0.001, $\eta_{p}^{2}$ = 0.88, reflecting a pattern of negative < neutral < positive pictures (all *p* < 0.001). The analysis on rating results of arousal revealed that the main effect of valence was significant, *F*(2, 18) = 57.43, *p* < 0.001, $\eta_{p}^{2}$ = 0.76, reflecting more arousing ratings for positive and negative pictures than for neutral pictures (all *p* < 0.001), with no significant differences between positive and negative pictures (*p* = 0.32). The main effect of group and interaction between valence and group were not significant.

For the 27 categories per valence, 18 were randomly selected to serve as studied categories. Specifically, for each of these categories, four exemplars were randomly presented at study and then served as old items at test; the remaining four exemplars were only presented as lures at test. The recognition status of each picture was determined randomly and then counterbalanced such that each picture set equally often served as old and lure items. The remaining nine categories served as unstudied categories, for which all exemplars were presented as new items at test.

The formal experiment was divided into three blocks, each including a study phase, a distracter task, and a test phase. The order of individual study-test blocks was counterbalanced across participants. Each study list included 24 pictures from six studied categories for each type of emotional valence. Each test list included 24 old pictures, 24 lure pictures from studied categories, and 24 new pictures from three unstudied categories for each valence condition. The positive, neutral, and negative pictures were equally represented in each block. The pictures were pseudo-randomized and presented with no more than three pictures from the same type of pictures (e.g., item type, valence, or category) presented consecutively at study and test. In addition, there were three primacy and three recency buffers separately presented at the beginning and end of the study and test phases in each block. Each participant completed a brief study-test practice session to familiarize themselves with the procedure before beginning the formal experiment. None of the practice stimuli appeared during the subsequent study or test phases.

### Procedure

Participants were seated individually at a viewing distance of approximately 100 cm from a computer monitor (1,080 × 1,024 pixels, 60 Hz refresh rate) controlled by a Lenovo PC and performed a categorized pictures task. After a brief practice session, participants undertook the experiment. The experiment was designed using E-Prime 2.0 software (Psychology Software Tools, Pittsburgh, PA). All stimuli were presented in the center of a black background on the computer monitor. The pictures subtended at a visual angle of 7° × 6.3°. Behavioral responses in both the study and the test phase were made using a computer keyboard.

At study, a 500-ms fixation cross (+) was presented in the center of the screen, followed by a 1,000-ms picture. Participants were required to remember the pictures for a subsequent test. Then, a rating interface was presented while participants were given 5,000 ms to rate the valence of the picture using a scale ranging from 1 (most negative) to 5 (most positive). The trial ended with a 500-ms blank screen. Following the study phase, a 120-s distracter arithmetic task was administered: participants counted backward by threes from 200. At test, the 500-ms fixation cross preceded a test picture with a maximum presentation time of 2,000 ms. Participants were required to indicate whether the picture was old or new *via* the computer keyboard, using the left or right index finger. Then, a confidence rating interface was presented with a maximum presentation time of 5,000 ms after a response was made, prompting the participants to assign a confidence rating to their recognition decision on a 5-point scale (1 = least confident, 5 = most confident). The inter-trial interval was 500 ms.

Participants were instructed to respond as fast and accurately as possible during old/new recognition. The computer key-response hand mappings were counterbalanced. Half of the participants made their responses of “old” by pressing the key “F” with their left index finger, and of “new” by pressing the key “J” using their right index finger. The other half responded “old” by pressing the key “J” using their right index finger, and “new” by pressing the key “F” using their left index finger.

## Results

### Neuropsychological Test Performance

The results of all neuropsychological tests are summarized in Table 1. Compared with young adults, older adults exhibited poorer performance on the trail making, digit span, block design, logic memory, and paired-association learning tests. In contrast, the older adults exhibited similar performance to the young adults on the vocabulary and fluency tests. These results indicate that aging is associated with a decline in fluid intelligence and preserved crystalized intelligence.

### Task Performance

All ANOVAs applied the Greenhouse–Geisser correction for non-sphericity of data when necessary. The uncorrected degrees of freedom, corrected *p*’s, and effect sizes ($\eta_{p}^{2}$) are reported. The *p*’s were adjusted using the Bonferroni correction for the pairwise comparisons. For all analyses, a significance level of 0.05 was adopted. All data were collapsed across confidence levels.

#### Valence Ratings

Valence ratings during the study phase were analyzed by a 3 (valence: positive vs. neutral vs. negative) × 2 (group: young vs. older) repeated measures ANOVA. There was a significant main effect of valence, *F*(2,102) = 279.50, *p* < 0.001, $\eta_{p}^{2}$ = 0.85, reflecting a pattern of negative [mean (SD) = 2.259 (0.417)] < neutral [3.214 (0.481)] < positive [3.734 (0.537)] pictures (all *p*’s < 0.001). This pattern corresponded with the pilot valence ratings to each picture valence. There was no significant main effect of group or interaction between valence and group.

#### False Recognition

**Figure 2:**
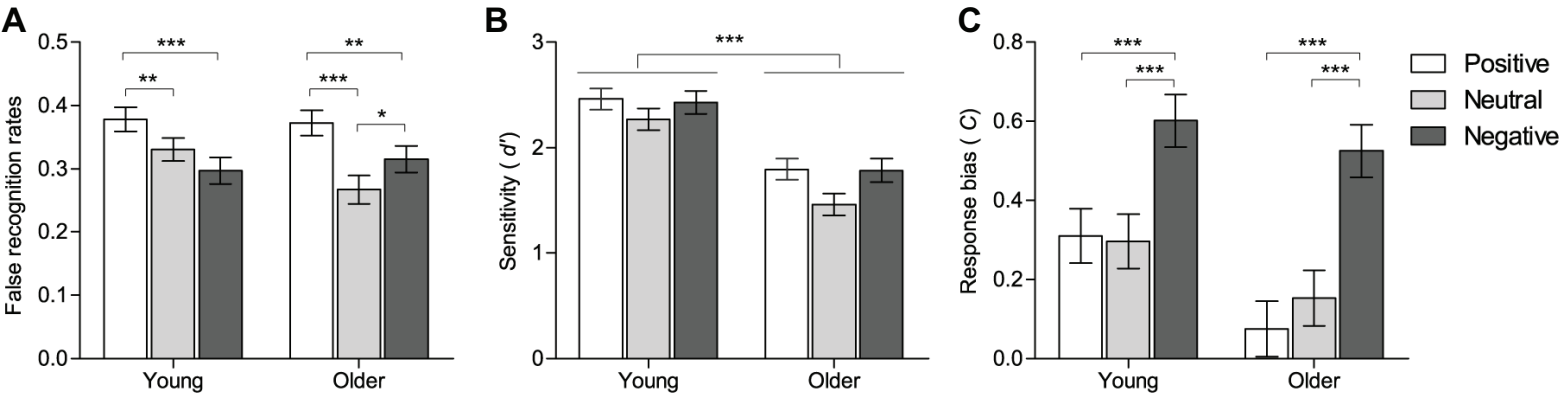
False recognition rates **(A)**, memory sensitivity **(B)**, and response bias **(C)** as a function of emotional valence for each age group. Error bars represent the standard error of the mean. *p < 0.05. **p < 0.01. ***p < 0.001.

Table 2 shows the mean proportions of old responses to old, lure, and new items as a function of emotional valence in young and older adults. Following Budson et al. (2006), false recognition performance was indexed by subtracting false alarm rates to new pictures (i.e., baseline false alarm rates) from false alarm rates to lure pictures. False recognition rates as a function of emotional valence and age group are displayed in Figure 2A.

**Table 2:** Mean proportions of old responses to old items, lures, and new items as a function of Valence in young and older adults (standard error of the mean).

Group	Item type	Valence		
		Positive	Neutral	Negative
Young	Old items	0.806 (0.021)	0.784 (0.026)	0.715 (0.024)
	Lure items	0.456 (0.024)	0.423 (0.027)	0.354 (0.024)
	New items	0.078 (0.017)	0.093 (0.015)	0.057 (0.014)
Older	Old items	0.776 (0.022)	0.695 (0.026)	0.635 (0.025)
	Lure items	0.563 (0.025)	0.477 (0.028)	0.412 (0.024)
	New items	0.191 (0.018)	0.210 (0.015)	0.097 (0.014)

A 3 (valence: positive vs. neutral vs. negative) × 2 (group: young vs. older) repeated measures ANOVA revealed a significant main effect of valence, *F*(2,102) = 27.19, *p* < 0.001, $\eta_{p}^{2}$ = 0.35, and a significant valence by group interaction, *F*(2,102) = 6.52, *p* = 0.002, $\eta_{p}^{2}$ = 0.11. The interaction was decomposed by conducting pairwise comparisons separately for each type of emotional valence. Young adults exhibited higher false recognition rates compared with older adults for neutral pictures (*p* = 0.029). There were no significant group differences for positive and negative pictures.

Pairwise comparisons were also conducted separately for each age group. Young adults showed higher false recognition rates for positive than for negative (*p* < 0.001) and neutral pictures (*p* = 0.009). False recognition rates for neutral and negative pictures did not differ significantly. Older adults showed higher false recognition rates for positive than for negative (*p* = 0.002) and neutral pictures (*p* < 0.001), and higher false recognition rates for negative than for neutral pictures (*p* = 0.023). These results revealed that both young and older adults exhibited higher false recognition rates for positive than for negative lures. To examine whether there is an age-related positivity effect in false recognition, we conducted a planned between-group comparison on the mean differences in false recognition rates between positive and negative valence (positive minus negative). Unfortunately, the results revealed no significant group differences. These findings suggest that older and young adults showed equivalent positive processing preferences and older adults did not show a positivity effect in false recognition memory.

#### True Recognition

Memory sensitivity [*d′ = z*(hits) − *z*(false alarms to new items)] was calculated for each valence condition in young and older adults according to signal detection theory (see Figure 2B; Macmillan and Creelman, 2004). Higher values of *d′* indicate greater sensitivity. A 3 (valence: positive vs. neutral vs. negative) × 2 (group: young vs. older) ANOVA revealed no significant interaction between valence and group. There was a significant main effect of group, *F*(1, 51) = 31.20, *p* < 0.001, $\eta_{p}^{2}$ = 0.38, revealing that older adults exhibited poorer performance on true recognition compared with young adults regardless of emotional valence. There was also a significant main effect of valence, *F*(2,102) = 9.87, *p* < 0.001, $\eta_{p}^{2}$ = 0.16, reflecting higher memory sensitivity for positive and negative compared with neutral pictures (all *p*’s = 0.001), with no significant differences between positive and negative pictures, regardless of age. Both young and older adults exhibited equivalent memory sensitivity for positive and negative pictures, suggesting that older adults did not display a positivity effect on memory sensitivity.

#### Response Bias

Response bias {*C* = −[*z*(Hits) + *z*(false alarms to new items)]/2} was calculated for valence condition in young and older adults according to signal detection theory (see Figure 2C). Lower values of *C* indicate more liberal response bias. A 3 (valence: positive vs. neutral vs. negative) × 2 (group: young vs. older) ANOVA revealed a significant main effect of valence, *F*(2,102) = 64.76, *p* < 0.001, $\eta_{p}^{2}$ = 0.56, reflecting that positive and neutral pictures were associated with a more liberal response bias compared with negative pictures (all *p*’s < 0.001). These results suggest that both age groups are more likely to endorse positive pictures as being recognized relative to negative pictures.

#### Confidence Ratings

Table 3 shows the mean confidence ratings assigned to old responses to old items (true recognition) and lures (false recognition) for positive, neutral, and negative pictures in both age groups. A 2 (response category: true vs. false recognition^3^) × 3 (valence: positive vs. neutral vs. negative) × 2 (group: young vs. older) repeated measures ANOVA revealed a significant response category by group interaction, *F*(1,51) = 60.47, *p* < 0.001, $\eta_{p}^{2}$ = 0.54. Pairwise comparisons showed that older adults displayed the same confidence during false recognition as during true recognition, whereas young adults were more confident during true recognition than false recognition (*p* < 0.001). In addition, older adults demonstrated higher levels of confidence than young adults during false recognition (*p* < 0.001), whereas age differences were not significant in confidence ratings when correctly recognizing old items. These results suggest that older adults exhibit greater susceptibility to high-confidence memory distortions, consistent with previous studies (Shing et al., 2009; Fandakova et al., 2013).

**Table 3:** Mean confidence ratings assigned to true and false recognition for positive, neutral, and negative pictures in young and older adults (standard error of the mean).

Group	Response category	Valence		
		Positive	Neutral	Negative
Young	True recognition	4.333 (0.085)	4.325 (0.090)	4.432 (0.072)
	False recognition	3.607 (0.111)	3.561 (0.110)	3.763 (0.117)
Older	True recognition	4.468 (0.087)	4.462 (0.092)	4.569 (0.073)
	False recognition	4.355 (0.113)	4.343 (0.112)	4.478 (0.119)

### Correlation Analysis

Finally, correlation analyses with age, gender, and education level as covariates were conducted separately for young and older adults to explore whether executive functioning was positively associated with positive processing preferences during false recognition.

**Figure 3:**
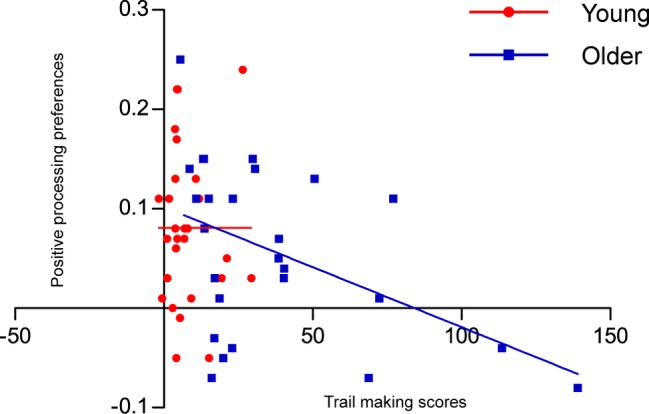
Scatterplot of the relationship between trail making scores (Trail Making B minus Trail Making A) and positive processing preferences in false recognition rates (positive minus negative) in young and older adults.

First, executive functioning was quantified by subtracting the scores on Trail Making A from the scores on Trail Making B. Lower trail making scores indicate higher executive functioning levels. Positive processing preferences were quantified by subtracting the false recognition rates for negative pictures from those for positive pictures. The results revealed that trail making scores were negatively correlated with the positive processing preferences in false recognition rates in older adults, *r* = −0.481, *p* = 0.02, but not in young adults (see Figure 3). These findings suggest that older adults with higher executive functioning are associated with greater positive processing preferences during false recognition.

In addition, it has been suggested that the central executive component of working memory plays an important role in the performance of Digit Span tests (Hester et al., 2004). As a result, we also conducted correlation analyses between positive processing preferences during false recognition and scores for Digit Span Forward and Digit Span Backward tests, respectively. The results indicated no significant correlations of positive processing preferences with Digit Span scores in both young and older adults.

As no significant differences between positive and negative pictures were revealed for memory sensitivity, we did not conduct correlation analyses between scores on executive functioning tests and positive processing preferences for true recognition performance.

## Discussion

Age-related changes in goal priorities may impact cognitive processing in a top-down manner. Older adults appear to prioritize their emotion regulation goals and show a positivity effect in memory. The present study was designed to examine age-related differences in false recognition rates for emotionally positive versus negative stimuli, and the role of executive functioning in older adults’ positive preferences in false recognition memory. Young and older participants studied emotional pictures from various categories during encoding and subsequently asked to make an old/new recognition judgment on the old, lure, and new pictures. Executive functioning was assessed using the Trail Making and Digit Span tests. We found that young and older adults differed in the false recognition rates for neutral pictures, whereas false recognition rates for negative and positive pictures were equivalent in the two age groups. In addition, both young and older adults showed preferences for positively valenced over negatively valenced lure pictures in false recognition. Interestingly, trail making scores were negatively associated with positive processing preferences in false recognition rates in older but not young adults. The implications of these findings for emotional aging are discussed below.

Episodic memory is regarded as a constructive process that may lead to memory distortions (Schacter et al., 1998). The production of false memory reflects the operation of adaptive rather than defective processes (Schacter and Addis, 2007). In the present study, we found significant group differences in the false recognition rates for neutral pictures but not for positive and negative pictures, suggesting that older adults exhibit equivalent adaptive memory processes for emotional pictures compared to young adults. These findings may reflect the fact that emotional pictures (especially negative ones) are more distinctive than neutral pictures and thus are less susceptible to the negative effects of aging. Interestingly, it seems that divided attention at encoding has similar effects as aging on the memory of emotional versus neutral stimuli (see Rossi-Arnaud et al., 2018, for discussion). For example, Maddox et al. (2012) found that divided attention at encoding reduced item and associative memory for neutral stimuli more than memory for negative stimuli.

The familiarity process underlies false memories by retrieving gist memory traces (Bookbinder and Brainerd, 2016). As people age, they may exhibit a tendency to rely on gist processing (Koutstaal and Schacter, 1997; Tun et al., 1998) and familiarity-based retrieval in recognition memory (Yonelinas, 2002; Koen and Yonelinas, 2014). Thus, we reasoned that older participants would falsely endorse more positive than negative lure pictures as “old” if their motivational changes toward emotion regulation. Consistent with our hypothesis, the results revealed older participants’ preference for positive over negative lures in their false recognition rates. These findings suggest that older adults may distort their memories in an emotionally gratifying way. These results are consistent with a previous study in which free-recall paradigm was used (Fernandes et al., 2008). However, in the present study, age differences in false memory for emotionally positive versus negative lure pictures were not significant, as young participants showed an equivalent positive processing preference over negative lure pictures in their false memory. Thus, we cannot conclude that older adults show a positivity effect on false memory. The potential reasons for young participants’ processing preference for positive over negative lure pictures are discussed as follows.

First, pictures rather than words were used as experimental stimuli in the present recognition memory task. It has been suggested that emotional pictures are more distinctive and arousing than emotional words (Choi et al., 2013). In particular, negative rather than positive pictures are usually remembered with more item-specific details (Ochsner, 2000; Johansson et al., 2004; Inaba et al., 2007). According to fuzzy-trace theory, item-specific details can greatly counteract the production of false memory (Bookbinder and Brainerd, 2016). Thus, it is possible that item-specific details of negatively valenced pictures suppress false recognition of lures during retrieval in young adults. This explanation is partially consistent with the present findings that young and older adults show different patterns of false recognition rates for negative and neutral pictures. It is well established that memory for item-specific details is dependent upon the recollection process, which is typically impaired in older adults (Koen and Yonelinas, 2014). As a result, the remembering of item-specific details for negative pictures may be less efficient in older adults. Accordingly, older adults display higher false recognition rates for negative than for neutral pictures in the present study. Interestingly, your adults show comparable false recognition rates for negative and neutral pictures, probably because they to a greater extent suppress false recognition of negative lures by retrieving item-specific details. Second, gist traces become enhanced as the number of exemplars presented per category is increased at encoding, and accordingly, the age differences in false memories are magnified under the larger category size condition (Koutstaal and Schacter, 1997; Paige et al., 2016). Consequently, the absence of significant age differences in the positive processing preferences in false recognition may be due to the use of a relatively small category size in the present study. Third, young adults also show a preference for positive over negative information in memory, if prompted to focus on their current emotional states (Mather and Johnson, 2000; Kennedy et al., 2004). In the current study, all participants were asked to rate the valence of pictures presented during encoding, which might, to some extent, motivate young participants to prioritize their emotional goals. Future research is needed to examine these speculations.

According to socioemotional selectivity theory, the age-related positivity effect in memory is derived from motivational changes to emotion regulation goals (Carstensen et al., 1999; Carstensen, 2006). Although emotion regulation goals are automatically activated in older adults, executive control processes are necessary for them to be able to implement their goals (Mather and Carstensen, 2005; Mather, 2006; Reed and Carstensen, 2012). Consistent with this proposal, our results revealed that older adults with better executive functioning (i.e., lower trail making scores) were associated with greater preferences for positive over negative lures in false recognition. These findings are in line with previous studies, in which executive functioning is shown to modulate positive processing preferences in memory in older adults (Mather and Knight, 2005; Petrican et al., 2008). The present study provides converging evidence that older adults, to a greater extent, distort emotionally positive information in an emotional goal-consistent way when more cognitive resources are available.

We did not find significant correlations between Digit Span scores and positive processing preferences in false recognition memory in older adults. A potential reason may be that different dimensions of executive functioning underlie trail making and Digit Span tests. The former is more likely to recruit executive abilities of set shifting and cognitive flexibility, whereas the latter may mainly involve active manipulation of the information held in the working memory (Tamez et al., 2011). The present findings suggest that trail making performance may be a more sensitive indicator for cognitive control abilities which play important roles in positive processing preferences in older adults. Nevertheless, more executive functioning tasks should be administered to enhance the generality of the association between positive processing preferences in false recognition and executive functioning in older adults in future studies.

With regard to true recognition performance, although memory sensitivity was better for young adults compared with older adults regardless of valence, we found that both young and older adults are more likely to remember emotional pictures than neutral ones. These findings are consistent with previous studies showing that emotional memory enhancement effect is preserved in aging (Kensinger et al., 2014). In addition, both young and older adults exhibited equivalent memory sensitivity for positive and negative pictures, suggesting that older adults did not show a positivity effect on memory sensitivity as reported in previous studies. The discrepancy may be associated with variations in the experimental paradigms. We utilized a false memory paradigm rather than traditional tests of recognition memory in the present study. It has been suggested that older adults exhibit the positivity effect in true memory because they implement their emotional regulation goals by strengthening positive and diminishing negative stimuli in memory (Carstensen, 2006; Mather, 2006). In the present study, it is possible that older adults are less likely to implement their emotional regulation goals on studied items (e.g., diminishing negative stimuli in true memory) as multiple exemplars per category are presented during encoding. Future research is needed to examine this speculation.

Overall, the contributions of the present study to the literature on emotional aging are reflected in three aspects. First, our current study explored the question of whether there would be an age-related positivity effect using a categorized pictures task specifically aimed to assess false recognition memory. Second, as noted before, compared with DRM paradigm used in Piguet et al. (2008), the present manipulations greatly eliminate the potential confounding of conceptual incongruence between the emotional lures and the study items on false recognition performance. Though false recognition memory exhibits preferences toward positively valenced stimuli in both young and older adults, it is reasonable to speculate that the age-related positivity effect would be revealed when larger category size is used in future studies. Third, the present findings greatly extended previous research by showing older adults’ positive association of executive functioning with their positive processing preferences in emotional false memory.

Several limitations should be noted. First, a relatively small category size (four exemplars) was used for the categorized picture paradigm in the current study. Future research is needed to explore whether older relative to young adults exhibit greater positive preferences in false recognition memory under the larger category size condition. Second, we only conducted pilot ratings of the two dimensions of emotion for picture materials. Although the number of picture exemplars was matched within each category across the three valence conditions, it is hard to guarantee that other features (e.g., visual similarity between picture exemplars) were fully matched for each valence condition in the current study. It is therefore necessary to develop a normative database for categorized pictures in the future. Third, in the present study, we only found significant correlations of positive processing preferences in false recognition memory with the trail making scores. Conclusions would be strengthened by the use of a broad battery of executive functioning tests in future studies.

## Conclusions

We investigated age differences in memory preferences for positive over negative lure pictures in false recognition memory using a categorized pictures paradigm. We observed that older and young adults exhibited equivalent positive processing preferences in false recognition. Executive functioning is associated with the preferential processing of positive versus negative lure pictures in older but not young adults. The present findings provide new evidence for motivational perspective in emotional aging and have important implications for the understanding of the interaction between cognition and emotion in older adults.

## Ethics Statement

This study was carried out in accordance with the recommendations of Ethics Committee of the Institute of Psychology, Chinese Academy of Sciences with written informed consent from all subjects. All subjects gave written informed consent in accordance with the Declaration of Helsinki. The protocol was approved by the Ethics Committee of the Institute of Psychology, Chinese Academy of Sciences.

## Author Contributions

JL conceived the idea and participated in the writing and revision of the manuscript. ZZ conceived the idea, analyzed and interpreted the data, and drafted and revised the manuscript. ML, WW, FX, and SG assisted the collection, analysis, and interpretation of data.

### Conflict of Interest Statement

The authors declare that the research was conducted in the absence of any commercial or financial relationships that could be construed as a potential conflict of interest.

## Footnotes

^1^Based on Reed et al. (2014), the positivity effect is operationally defined as a Group × Valence interaction in true or false recognition task. Specifically, older relative to young adults exhibit greater positive processing preferences. Here, positive processing preferences refer to higher true or false memory performance for positive versus negative stimuli.

^2^Specifically, we first obtained the category names for each type of emotional valence (e.g., cake, grave, and street scene). And then, we used Baidu Image search (https://image.baidu.com/) to select the categorized pictures based on these category names.

^3^Only the incorrect old responses to lures were included in the false recognition category.
